# Growing together gives more rice and aquatic food

**DOI:** 10.7554/eLife.77202

**Published:** 2022-02-22

**Authors:** Jian Liu, Siri Caspersen, Jean WH Yong

**Affiliations:** 1 School of Environment and Sustainability, Global Institute for Water Security, University of Saskatchewan Saskatoon Canada; 2 Department of Biosystems and Technology, Swedish University of Agricultural Sciences Alnarp Sweden

**Keywords:** paddy ecosystem, aquatic animals, nitrogen recycling, facilitation, co-culture, sustainable cultivation, aquaculture, Other

## Abstract

Allowing aquatic organisms to grow in rice fields – a practice called co-culture – increases rice yields while maintaining soil fertility and reducing weeds.

**Related research article** Guo L, Zhao L, Ye J, Ji Z, Tang J, Bai K, Zheng S, Hu L, Chen X. 2022. Using aquatic animals as partners to increase yield and maintain soil nitrogen in the paddy ecosystems. *eLife* **11**:e73869. doi: 10.7554/elife.73869.

When you eat rice with fish – or rice with crab or shrimp – you probably do not think about where the food came from. And if you do, you probably think that the rice grew in a paddy field, while the fish, crab or shrimp were caught in the sea. However, this may only be partially true. Systems for growing rice and various aquatic animals together have existed for over 1,200 years, but the practice of ‘co-culture’ has only recently gained the attention of the major rice-producing nations and the scientific community (Xie et al., 2011).

Rice is one of the most widely consumed grains in the world and is grown in more than 100 countries. It is a staple food source for over half of the world’s population and of upmost importance for lower income countries in Asia, Latin America and Africa (Bashir et al., 2020). Climate change, declining natural resources and an ever-growing population put immense pressure on both increasing yields and reducing the environmental footprint of rice (Hu et al., 2016; Ahmed and Turchini, 2021). Global trends are thus moving towards sustainable and organic management of biological resources (Chen et al., 2014; Muller et al., 2017). Strategic coupling of terrestrial and aquatic ecosystems, such as growing crops and aquatic animals together, could help meet this target (Ahmed and Turchini, 2021).

Previous research has shown that co-cultures can boost yields, improve soil health and enhance ecosystem services (Mueller et al., 2012; Campanhola and Pandey, 2019). But even though co-culture systems would help optimise the use of land and water resources to produce food – while reducing the environmental impacts associated with rice monocultures – large-scale and long-term data are lacking (Bashir et al., 2020).

Now, in eLife, Xin Chen and colleagues at Zhejiang University and Bioversity International – including Liang Guo and Lufeng Zhao as joint first authors – report new evidence in support of co-cultures with aquatic animals and rice crops (Guo et al., 2022). Between 2017 and 2020, the team conducted three separate field experiments in which rice was grown with either fish, crabs or soft-shelled turtles. Each set-up also included a control experiment, where rice was grown as a monoculture. No agrochemicals were used to control weeds, pests or diseases during the field trials.

Over the four years, the co-cultures demonstrated multiple benefits (Figure 1). Rice yield was consistently higher in fields containing aquatic animals (between 8.7% and 12.1%). Moreover, the team was also able to harvest significant amounts of fish, crab and turtle as food (between 560 and 2660 kg/ha). Co-cultures also had fewer weeds and maintained consistent levels of mineral nutrients (nitrogen and phosphorus) in the soil. Moreover, the breakdown of organic matter happened faster in the co-cultures.

**Figure 1.**
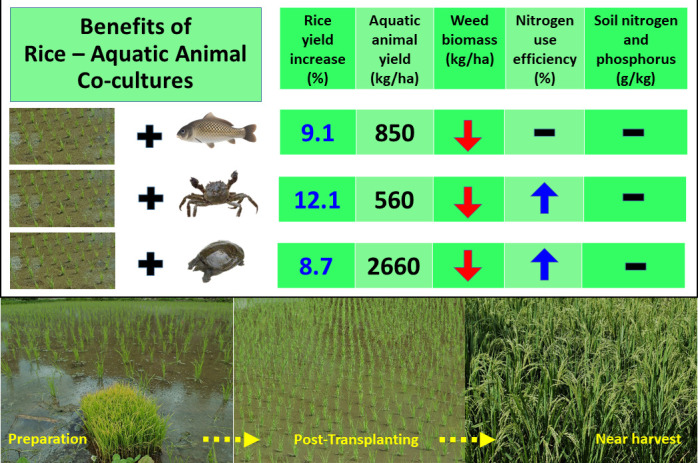
The benefits of co-culture for growing rice. Guo et al. showed that growing rice with aquatic animals (fish, crabs or turtles) increases rice yield, suppresses the growth of weeds, and maintains the levels of nitrogen and phosphorus in the soil. Growing rice with crabs or turtles was also shown to promote a more efficient use of nitrogen. The photographs show the field before (left) and after (middle) the rice plants were transplanted, and near harvest time (right). The aquatic animals were introduced as juveniles about a week after transplanting and lived with the rice plants throughout the experimental periods.

Animals are instrumental in moving elements, such as carbon, nitrogen and phosphorus, in the environment (Schmitz et al., 2018). To find out whether the biology of a co-cultured animal would affect the growth of rice, Guo et al. carried out three additional, controlled experiments to trace the movement of nitrogen from feed (labelled with stable isotopes) to aquatic animals and the environment.

Analyses of the animal’s food intake revealed that fish and crabs obtained up to half of their diet (50% and 35%, respectively) from the rice fields, consuming algae, phytoplankton or weeds. Turtles relied more on additional feed, and only derived 16% of their food intake naturally. The animals’ wastes and any uneaten feed also increased the nutrient availability for the rice plants: rice plants used up to a third of the nitrogen from the animal feed.

The work of Guo et al. demonstrates clearly how co-cultures could make agriculture more sustainable, by increasing soil fertility and reducing the need for fertilizers or pesticides. Moreover, these coupled systems could also help fight the spread of malaria by introducing natural, co-culturing predators, such as frogs (which eat the mosquitos) and fish (which eat the mosquito larvae), and so contribute towards several ‘Sustainable Development Goals’ of the United Nations (Khatiwada et al., 2016; Campanhola and Pandey, 2019).

More research is needed to better understand the impact of co-culture on greenhouse gas emissions and nutrient pollution (Bashir et al., 2020). Nevertheless, these experiments provide a good foundation for further studies to explore how agriculture can be made more sustainable.

## References

[bib1] Ahmed N, Turchini GM (2021). The evolution of the blue-green revolution of rice-fish cultivation for sustainable food production. Sustainability Science.

[bib2] Bashir MA, Liu J, Geng Y, Wang H, Pan J, Zhang D, Rehim A, Aon M, Liu H (2020). Co-culture of rice and aquatic animals: An integrated system to achieve production and environmental sustainability. Journal of Cleaner Production.

[bib3] Campanhola C, Pandey S (2019). Sustainable Food and Agriculture: An Integrated Approach.

[bib4] Chen X, Cui Z, Fan M, Vitousek P, Zhao M, Ma W, Wang Z, Zhang W, Yan X, Yang J, Deng X, Gao Q, Zhang Q, Guo S, Ren J, Li S, Ye Y, Wang Z, Huang J, Tang Q, Sun Y, Peng X, Zhang J, He M, Zhu Y, Xue J, Wang G, Wu L, An N, Wu L, Ma L, Zhang W, Zhang F (2014). Producing more grain with lower environmental costs. Nature.

[bib5] Guo L, Zhao L, Ye J, Ji Z, Tang J, Bai K, Zheng S, Hu L, Chen X (2022). Using aquatic animals as partners to increase yield and maintain soil nitrogen in the paddy ecosystems. eLife.

[bib6] Hu LL, Zhang J, Ren WZ, Guo L, Cheng YX, Li JY, Li KX, Zhu ZW, Zhang JE, Luo SM, Cheng L, Tang JJ, Chen X (2016). Can the co-cultivation of rice and fish help sustain rice production?. Scientific Reports.

[bib7] Khatiwada JR, Ghimire S, Khatiwada SP, Paudel B, Bischof R, Jiang J, Haugaasen T (2016). Frogs as potential biological control agents in the rice fields of Chitwan, Nepal. Agriculture, Ecosystems & Environment.

[bib8] Mueller ND, Gerber JS, Johnston M, Ray DK, Ramankutty N, Foley JA (2012). Closing yield gaps through nutrient and water management. Nature.

[bib9] Muller A, Schader C, El-Hage Scialabba N, Brüggemann J, Isensee A, Erb KH, Smith P, Klocke P, Leiber F, Stolze M, Niggli U (2017). Strategies for feeding the world more sustainably with organic agriculture. Nature Communications.

[bib10] Schmitz OJ, Wilmers CC, Leroux SJ, Doughty CE, Atwood TB, Galetti M, Davies AB, Goetz SJ (2018). Animals and the zoogeochemistry of the carbon cycle. Science (New York, N.Y.).

[bib11] Xie J, Hu LL, Tang JJ, Wu X, Li NN, Yuan YG, Yang HS, Zhang JE, Luo SM, Chen X (2011). Ecological mechanisms underlying the sustainability of the agricultural heritage rice-fish coculture system. PNAS.

